# Antiproliferative activities of some selected Nigerian medicinal plants against breast, liver, and cervical cancer cells

**DOI:** 10.1186/s12906-024-04365-w

**Published:** 2024-03-06

**Authors:** Olubusola O. Olaleye, Dong-Hyun Kim, Keith A. Spriggs

**Affiliations:** 1 School of Pharmacy, University of Nottingham, University Park, Nottingham, NG7 2RD UK; 2 Department of Pharmacognosy, Faculty of Pharmacy, University of Lagos, Lagos State, Nigeria

**Keywords:** Medicinal plants, Phytochemicals, Cytotoxicity, Polyphenolic content, Chemotherapeutic agent

## Abstract

**Background:**

Phytochemicals have become a growing source of alternative medicine in developing countries due to the poor prognosis, high cost of conventional pharmaceuticals, and undesirable effects associated with mainstream cancer treatment.

**Objective:**

This study was aimed at investigating the anticancer effect of some selected Nigerian medicinal plants used in cancer treatment. These include ethanol extracts of *Dialium guineense* root (DGR), *Dialium guineense* leaves (DGL), *Jateorhiza macrantha* leaves (JML), *Musanga cecropioides* leaves (MCL), *Musanga cecropioides* stembark (MCSB), *Piptadeniastrum africanum* stembark (PASB), *Piptadeniastrum africanum* root (PAR), *Pupalia lappacea* flower tops (PLF), *Raphiostylis beninensis* root (RBR), *Raphiostylis beninensis* leaves (RBL), *Ritchiea capparoides* leaves (RCL), *Ritchiea capparoides* stembark (RCSB), and *Triplochiton scleroxylon* stembark (TSB).

**Methods:**

The cytotoxic activity of the extracts was examined using a brine shrimp lethality assay and 3-(4,5-dimethylthiazol-2-yl)-2,5-diphenyltetrazolium bromide (MTT) assay against three cancer cell lines, including MCF-7, HUH-7, and HeLa. The selectivity of all extracts towards cancer cells was investigated using normal lung fibroblasts (MRC-5). Cell migration and colony-forming assays of active extracts against MCF-7 cells were also performed. Additionally, the total polyphenolic contents of the active extracts were estimated using standard methods.

**Results:**

The extract of PASB had the highest cytotoxicity (LC_50_ = 1.58 μg/mL) on the brine shrimps compared to vincristine sulphate (LC_50_ = 2.24 μg/mL). In the cell viability assay, all the extracts produced significant (*p* < 0.05) growth inhibitory effects against all cell lines tested in a dose-dependent manner. All extracts were selective to cancer cells at varying degrees. Worth mentioning are the extracts of MCL, DGR, RBR, and PASB, which exhibited 14-, 7-, 6- and 2-fold selectivity toward MCF-7 cancer cells relative to normal lung fibroblast (MRC-5), respectively. These four extracts also significantly inhibited cell migration and colony formation in MCF-7-treated cells in dose-dependent manners. Considerable amounts of phenolics, flavonoids, and proanthocyanidins were detected in all extracts evaluated.

**Conclusion:**

These findings advocate the continued development of MCL, DGR, RBR, and PASB as potential chemotherapeutic agents.

**Supplementary Information:**

The online version contains supplementary material available at 10.1186/s12906-024-04365-w.

## Introduction

In many parts of Africa, cancer death rates are on the increase due to unaffordable healthcare costs and lack of medical facilities [1]. Breast, cervical, prostate, colorectal, liver, and non-Hodgkin lymphoma are the most prevalent malignancies in Nigeria [2]. Chemotherapy, radiotherapy, and surgery are among the many treatment options, used singly or in combination, however, access to these is often limited, and adverse effects are common [3].

Given the untoward effects of chemotherapeutic interventions on healthy cells, there is a critical need to discover new targeted therapies that are safe and effective in cancer treatment [4]. Medicinal plants contain numerous compounds such as flavonoids, saponins, alkaloids, tannins, and phenolics that have proven therapeutic efficacy against a wide range of human diseases including cancer [5].

Natural products, particularly those from medicinal plants have played significant roles in the development of notable anticancer agents including vincristine, vinblastine, vinorelbine, and vindesine from *Catharanthus roseus*, paclitaxel from *Taxus baccata* and etoposide from *podophyllum peltatum* [6]. Despite this, many traditional medicinal plants have not been fully scientifically evaluated as potential anticancer therapeutic agents. A wide literature search on plants used in folk medicine for the management of tumours and tumour-related problems such as pain, inflammation, and oedema in Nigeria and West Africa was undergone using Web of Science, Scopus, PubMed, and JSTOR databases.

From these results, thirteen extracts from eight indigenous plants with information on the cytotoxicity profile but with little or no information on the active compounds were selected. The extracts from the plants include *Dialium guineense* root (DGR), *Dialium guineense* leaves (DGL), *Jateorhiza macrantha* leaves (JML), *Musanga cecropioides* leaves (MCL), *Musanga cecropioides* stembark (MCSB), *Piptadeniastrum africanum* stembark (PASB), *Piptadeniastrum africanum* root (PAR), *Pupalia lappacea* flower tops (PLF), *Raphiostylis beninensis* root (RBR), *Raphiostylis beninensis* leaves (RBL), *Ritchiea capparoides* leaves (RCL), *Ritchiea capparoides* stembark (RCSB), and *Triplochiton scleroxylon* stembark (TSB). Although preliminary cytotoxic information of some of these plants exists [7–10], there is little evidence on the isolation and characterization of the bioactive compounds. These studies also used only brine shrimps lethality assay, one cancer cell line, one cell proliferation assay (3-[4,5-dimethylthiazol-2-yl]-2,5 diphenyl tetrazolium bromide, (MTT)), and did not consider the safety profile of these extracts on the non-cancer cell line. We, therefore, focused on expanding the cytotoxic activity of the different parts of the selected plants using a panel of cancer cell lines (MCF-7, HUH-7, HeLa cells) along with more assay methods (cell count, clonogenic, and cell migration assays). We investigated the selectivity of the extracts towards cancer cell lines relative to non-cancer cell line in a bid to identify extracts that are not only active but also safe on normal cells. This research is part of a broader study that will furnish us with information that will serve as the basis for identifying active extracts with promising bioactive principles upon which isolation and characterization processes can be performed.

In response to the folkloric utility of these plants and the search for effective and safe bioactive phytochemicals with cytotoxic properties, we investigated the ethanol extracts of these selected Nigerian medicinal plants as potential anticancer agents. The cytotoxic activity of these plant extracts was tested on three human cancer cell lines including MCF-7 (breast carcinoma), HUH-7 (liver carcinoma), HeLa (cervical carcinoma), and non-cancer cells, human foetal lung fibroblast (MRC-5). Cytotoxicity studies employed include brine shrimp lethality, MTT (3-(4, 5-dimethylthiazol-2-yl)-2,5-diphenyltetrazolium bromide), cell count, clonogenic and cell migration assays.

## Materials and methods

### Plant selection

The information about ethnomedicinal plants used in Nigeria for cancer therapy came from peer-reviewed publications and books on the subject, Table 1.

**Table 1:** Plants selected for the study

S/no	Plant/ plant parts	Ethnopharmacological uses	Common name/Local name	Location of collection and Coordinates	Voucher number	References
1.	*Dialium guineense* Wild (Leguminosae) Leaves and root	Leaves, root, and stem-bark are used as remedies for tumours	Velvet tamarind/ Awin	Moniya Ijaye road, Ibadan, Oyo state Latitude: 7.5237°N, Longitude: 3.9147°E	FHI 113110	[20–22]
2.	*Musanga cecropioides* R.Br & Tedlie (Urticaceae) Leaves and stem bark	Leaves and stem-bark decoctions are used to treat hard abscess	English umbrella tree/ Agbàwọ̀	Oru Ijebu, Ijebu Igbo, Ogun state Latitude: 6.9526° N, Longitude: 3.9434° E	FHI 113104	[23]
4.	*Piptadeniastrum africanum* (Hook.f.) Brenan. (Mimosaceae) Root and stem-bark	Powdered leaf, root, and stem bark in palm wine is applied topically on tumours	African Greenheart/ Odan	Onigambari Forest Reserve, Ibadan, Oyo state Latitude: 7° 25′ and 7° 55′N Longitude: 3° 53′ and 3° 9′E	FHI 113107	[24, 25]
5.	*Triplochiton scleroxylon* Schumann (Sterculiaceae) Stem-bark	The plant is used in traditional medicine to treat oedema and as an analgesic.	African whitewood/Obeche	Onigambari Forest Reserve, Ibadan, Oyo state Latitude: 7° 25′ and 7° 55′N Longitude: 3° 53′ and 3° 9′E	FHI 113106	[24]
6.	*Pupalia lappacea* (L) Juss. (Amaranthaceae) Leaves and flowers	The foliage is used in the form of poultices and decoctions to treat cancer	Ram’s bur/emọ́ àgbò	Agbegi village, Ikire, Osun state Latitude: 7° 21′ 36.00″ N Longitude: 4° 11′ 6.00″ E.	LUH 7680	[8]
7.	*Raphiostylis beninensis* Hook F. ex Planch (Metteniusaceae) Leaves and root	Crushed leaves and twigs are applied on glandular swellings.	Kpolokoto	Agbegi village, Ikire, Osun state Latitude: 7° 21′ 36.00″ N Longitude: 4° 11′ 6.00″ E.	FHI 113105	[10, 26, 27]
7.	*Ritchiea capparoides* Andr. Britten (Capparaceae) Leaves and stem bark	The powdered stem-bark is steeped in palm wine and drunk for swellings around the groin.	Shepherd’s banana/ lógbònkíyàn	Akure- Ondo Road Akure, Ondo state Latitude: 7° 15′ 25.6788″ N Longitude: 5° 12′ 20.8476″ E	FHI 113109	[28, 29]
8.	*Jateorhiza macrantha* (Hook.f.) Exell & Mendonça Leaves	Fresh leaves are applied topically on abscesses and breast tumours	Flat hand of monkey/ átatóbemẹ	Aramoko town, Aramoko, Ekiti state Latitude: 7° 42′ 17.39″ N Longitude: 5° 2′ 25.94″ E	FHI 113108	[30, 31]

### Collection of plants

Fresh plant samples were collected from the South-Western region of Nigeria in February 2021. Plant materials were collected after obtaining permissions from the Forestry Research Institute of Nigeria (FRIN) and taxonomical identifications were performed by Chukwuma Emmanuel of the same institute. The plants were assigned ascension numbers and voucher specimens were deposited at the Forestry Herbarium Ibadan (FHI) and Lagos University Herbarium (LUH). To conserve the plants, new plants were grown particularly those in which root parts were collected.

### Preparation of plant extract

Plant samples were dried under shade for 2 weeks and powdered using a laboratory blender (Christy and Morris 8** Lab Mill). Each powdered plant material was macerated with 96% ethanol for 72 hours. The resultant mixtures were decanted and filtered. The resultant filtrates were concentrated using a rotary evaporator (Buchi Rota-vapor R205, Brinkman, Switzerland) at 40 °C.

### Chemicals and reagents

All the chemicals and reagents used in this experiment were of analytical grade obtained from Sigma Aldrich. These include ethanol, dimethyl sulfoxide (DMSO), quercetin, vincristine sulphate, and MTT (3-(4,5-dimethylthiazol-2-yl)-2,5-diphenyltetrazolium bromide).

### Brine shrimp (*Artemia salina*) lethality bioassay

The toxicity of the selected crude extracts was conducted on freshly hatched brine shrimps. Briefly, 0.1 g of brine shrimps’ eggs (obtained from the Department of Pharmacognosy, University of Lagos, Nigeria) were placed in a hatching tray containing natural sea water obtained from Oniru beach, Lagos, Nigeria. Before usage, the tray was aerated and illuminated for 48 hours at 25 °C to guarantee the survival of the shrimps.

After hatching, ten brine shrimp larvae were put in tubes containing 5 mL of natural seawater. Extracts were tested at 10, 100, and 1000 μg/mL. Also, lower concentrations including 0.01, 0.1, and 1 μg/mL were used for one of the extracts, PASB (*n* = 3). After 24 hours, survivors were counted with the aid of a magnifying hand lens, and 50% lethal concentration (LC_50_) was determined using the probit analysis [11].

### Preparation of extract stock and working solution

Each extract (100 mg) was pre-solubilized in 1 mL DMSO giving stock solution of 100 mg/mL. This was filtered through a sterile filter of 0.20 μm before use. Working concentrations of 1000 μg/mL were prepared with DMEM from the stock solution and serial two-fold dilutions were prepared, giving test concentrations of 1.95–250 μg/mL. A lower concentration range between 0.001 to 10 μg/mL was also used for the extracts of PASB and PAR against the MCF-7 cell line due to the observed potent cytotoxic effect produced with the selected concentration range. In all the test concentrations, the DMSO final concentration was below 1%.

### Cell culture

The human cancer cells used for the study namely, MCF-7 (breast carcinoma), HUH-7 (liver carcinoma), and HeLa (cervical carcinoma) were obtained from the American Type Culture Collection (ATTC), Manassas, USA, and stored in liquid nitrogen tank at the tissue culture unit of Gene Regulation and RNA Biology Laboratory of the School of Pharmacy, University of Nottingham, United Kingdom while the non-tumorigenic lung fibroblast, MRC-5 cells were kindly provided by Dr. Tracey Bradshaw of Biomolecular Science and Medicinal Chemistry division, School of Pharmacy, University of Nottingham, United Kingdom. The cells were maintained in Dulbecco’s Modified Eagle’s Medium (DMEM) supplemented with 2 mM L-glutamine and 10% foetal calf serum (FCS) and were maintained in an incubator at 37 °C and 5% CO_2_ and were routinely sub-cultured twice weekly to maintain continuous logarithmic growth.

### The cell viability assay

The cytotoxic effects of the selected extracts were investigated on MCF-7, HUH-7, and HeLa (cervical) cancer cell lines. The medium was changed after incubation with a new medium containing various amounts of extracts or the vehicle. The final concentrations of the extracts were 1.95–250 μg/mL (or 0.001–10 μg/mL for PASB and PAR against the MCF-7 cell line). DMSO of corresponding concentrations was used as negative control while quercetin, a natural flavonoid was used as positive control, and the plates were then incubated for 72 h. At the time of treatment, cells were also planted in a time zero (T_0_) plate to measure viable cells. After incubation, 10 μL of 4 mg/mL MTT solution (in PBS) was added to each well and the plates were incubated for 4 h. DMSO (150 μL) was used to dissolve the formazan crystals formed after removing the medium. Plates were placed on a shaking table for 5–7 min and incubated for 15 min at 37 °C and then read using a BioTek Synergy HTX Multi-Mode Microplate Reader) at 560 nm. MTT assays were carried out in three independent experiments and the concentration that produced 50% growth inhibition, GI_50_ was calculated using the formula given below.

1 $$OD\ {GI}_{50}=\frac{Cont-{T}_0}{2}+{T}_0$$

2 $$OD\ {GI}_{50}=\frac{HOD- OD{GI}_{50}}{HOD- LOD}\ x\ \left( HC- LC\right)+ LC$$

Where: OD GI_50_ = Absorbance value of GI_50_; Cont = Absorbance value of Untreated; T_0_ = Absorbance value at time zero; HOD = high absorbance value where GI_50_ lies; LOD = low absorbance value where GI_50_ lies; HC = High Conc. value where GI_50_ lies; LC = Low Conc. value where GI_50_ lies) [12].

### Selectivity index (SI)

Human foetal lung fibroblast MRC-5 cells were used to investigate the selectivity of all the selected extracts toward cancer cells. The cells were grown in Minimum Eagle Medium supplemented with 10% foetal bovine serum (FBS), L-glutamine, non-essential amino acids, and HEPES solution (1 mM) kept in an incubator at 37 °C and 5% CO_2_. Briefly, MRC-5 cells (3 × 10^3^ cells/well) were treated with extracts at the same concentrations used with the cancer cell lines, and an MTT assay was performed after 72 h incubation. The SI was calculated as the ratio of the GI_50_ value on normal cell line to the GI_50_ on cancer (MCF-7, HUH-7, and HeLa) cell lines [13].

$$Selectivity\ index=\frac{GI_{50\ of\ MRC-5}}{GI_{50\ of\ MCF-7,\kern0.75em HUH-7\ or\ HeLa}}$$

### Cell counting assay

Cell counts were performed to corroborate MTT assay results. Cancer cells were seeded in 6 well plates at 2 × 10^4^ cells/well and incubated overnight. All extracts were added at concentrations of 1.95, 15.63, and 125 μg/mL (0.1, 1, and 10 μg/mL for PAR and PASB with MCF-7 cells only). Cells were harvested and counted with a hemocytometer after 72 h incubation [14].

### Selection criteria

To determine which extracts and cell lines to continue further studies on, some selection criteria were set. Criterion one: which extracts were active against at least two cell lines based on the American Cancer Institute recommendation of ≤30 μg/mL GI_50_ while also producing a dose-dependent inhibitory effect? Criterion two: Which cell line were the active extracts more selective to? Based on these criteria, four extracts including MCL, DGR, PASB, and RBR were chosen for further studies using the MCF-7 cancer cell line.

### Clonogenic assay

Clonogenic assays were conducted to evaluate the ability of single cells to survive a brief exposure to the test agent and maintain proliferative potential to form colonies [15]. To do this, MCF-7 cells were seeded in 6-well plates at 250 cells per well and treated with 0.5 x GI_50_, 1 x GI_50,_ and 2 x GI_50_ concentrations of the different plant extracts of MCL, DGR, RBR, and PASB obtained from the MTT assay and 0.1% DMSO (solvent control). After 24 h of incubation, the culture medium containing extracts was removed and was also replaced every three days. Experiments were terminated when colonies of greater than 50 cells were visible in control wells (approximately 10 days). All colonies were washed with PBS, fixed with methanol: acetic acid (3:1), and stained with 0.5% crystal violet followed by gentle washing with double distilled water. The plates were left to dry after which colonies were counted and images were taken with a digital camera.

### Cell migration assay

Inhibition of cell migration and metastasis was evaluated through a scratch assay on MCF-7 cells by a previously described method [16]. Concisely, cells (4 × 10^4^ cells/well) were seeded in 24 well plates which were cultured overnight. After incubation, a scratch was made with a 200 μL sterile pipette tip. The detached cells and other cellular debris were removed by washing with phosphate-buffered saline. Cells were treated with 1 x GI_50_ and 2 x GI_50_ concentrations of the different plant extracts (MCL, DGR, RBR, and PASB) obtained from the MTT assay and 0.1% DMSO (solvent control). The migration of cells was observed in the images taken by an inverted microscope (Olympus), equipped with a digital camera. The width of the scratch and wound closure at different time intervals (0, 24, 48, and 72 h) was analyzed by ImageJ software. The experiment was independently performed at least three times in triplicates. The migration rate (%) was calculated:

$$Migration\ rate=\frac{Wound\ area\ at\ 0\ h- wound\ area\ at\ 24\ or\ 48\ or\ 72\ h}{Control}$$

### Estimation of total phenolics, flavonoids and proanthocyanidins

In a bid to obtain information about some classes of bioactive compounds that play vital roles in cancer therapy, we quantified the amount of total phenolics, flavonoids, and proanthocyanidins in the active extracts. It is believed that results from this investigation will later guide the isolation and characterization process.

### Estimation of total phenolic content

The total phenolic content of the extracts was determined using Folin-Ciocalteau’s reagent, where gallic acid was used as a reference phenolic compound [17]. Gallic acid was prepared in methanol at five concentrations (0.01–0.05 mg/mL) and the plant extracts were also prepared in methanol at a concentration of 1 mg/mL. From each of the extract solutions, 0.5 mL was mixed with 2.5 mL of 1 in 10 dilutions of Folin-Ciocalteau’s reagent and 2 mL of 7.5% sodium carbonate. The extract was estimated at a final concentration of 0.1 mg/mL. The absorbance of the resulting blue colour solution was measured at 760 nm using a spectrophotometer after incubating the samples for 30 min at room temperature. All determinations were replicated at three different times (*n* = 3). The total phenolic contents were expressed as gallic acid equivalent (GAE) using the following equation based on the calibration curve: y = 26.344x + 0.093, R^2^ = 0.9926, where y is the absorbance and x is the gallic acid equivalent (mg/g).

### Estimation of total flavonoid content

The total flavonoid content was determined using the aluminium chloride method reported by [18] with slight modification. In brief, an ethanol solution of 2% AlCl_3_ (1.5 mL) was added to 1.5 mL of each extract sample (1.0 mg/mL in methanol). The mixture was incubated for 1 h at room temperature after which the absorbance was measured at 420 nm. The extract was evaluated at a final concentration of 0.1 mg/mL. A yellow colour indicated the presence of flavonoids. Quercetin, prepared in methanol at five different concentrations of 0.01–0.05 mg/mL was used as a reference flavonoid compound. Results were calculated as quercetin equivalent (mg/g) using the equation based on the calibration curve: y = 38.169x – 0.0327, R^2^ = 0.9957, where y is the absorbance and x is the catechin equivalent (mg/g). All determinations were replicated at three different times (*n* = 3).

### Estimation of total proanthocyanidin content

The total proanthocyanidin was estimated following the protocol by [19]. Concisely, 1 mL of prepared extracts (1 mg/mL) was mixed with 3 mL of 4% vanillin solution in methanol and 1.5 mL hydrochloric acid (1 N). The final concentration of the extract was 0.1 mg/mL. The mixture was allowed to stand for 15 minutes, and the absorbance was measured at 500 nm. The final concentration of the extract was 0.1 mg/mL. Total proanthocyanidin contents were calculated as catechin equivalent (mg/g) using the equation based on the calibration curve: y = 2.2923x – 0.0185, R^2^ = 0.9766, where y is the absorbance and x is the catechin equivalent (mg/g). All samples were analyzed in triplicate at three separate times (*n*=3).

### Statistical analysis

Results are presented as mean values ± standard deviation. GraphPad Prism Software version 9.4.1 (681) was used to compare several groups using a two-way ANOVA with Tukey’s post hoc test. Statistical significance was defined as a value of *p* < 0.05 and *p* < 0.0001.

## Results

### Plant selection

The plants selected for the study, including their ascension number are presented in Table 1.

### Yield of extracts

The extraction of the different parts of the selected plants resulted in thirteen extracts which were used for the study, Table 2.

**Table 2:** Amount and % yield of extracts

S/no	Extracts	Weight of powdered samples (g)	Amount of extracts (g)	Yield (w/w %)
1	DGL	1216	133	10.94
2	DGR	1272	103	8.1
3	JML	1426	61	4.28
4	MCL	3890	531.7	13.67
5	MCSB	2615	1500	57.36
6	PASB	1998	146	7.31
7	PAR	1583	43	2.72
8	PLF	1245	39	3.13
9	RCL	1246	71.6	5.75
10	RBL	1515	21	1.39
11	RBR	1083	38	3.51
12	RCSB	1386	48	3.46
13	TSB	1109	20	1.8

### Brine shrimps lethality assay

The brine shrimp lethality test was used to screen thirteen different extracts obtained from the selected plants. All the extracts exhibited LC_50_ values less than 1000 μg/mL except *Dialium guineense* leaves (DGL) and *Musanga cecropioides* stembark (MCSB). When compared to Vincristine sulphate (LC_50_ = 2.24 μg/mL), *Piptadeniastrum africanum* stembark (PASB) extract (LC_50_ = 1.58 μg/mL) exhibited the maximum cytotoxicity on brine shrimps, Table 3.

**Table 3:** LC_50_ of plant extracts determined by brine shrimp lethality assay

S/no	Extracts and standards	LC_50_ (μg/mL)
1	DGL	1778 ± 0.38
2	DGR	112.2 ± 0.58
3	JML	125.89 ± 0.58
4	MCL	199.53 ± 0.91
5	MCSB	1030 ± 1.46
6	PASB	1.58 ± 0.58
7	PAR	22.39 ± 0.72
8	PLF	39.8 ± 0.91
9	RCL	177.83 ± 0.86
10	RBL	135.48 ± 2.03
11	RBR	19.95 ± 0.72
12	RCSB	630.96 ± 0.86
13	TSB	112.2 ± 1.08
14	Vincristine sulphate	2.24 ± 0.96

Data are presented as mean ± SD of three different experiments performed in triplicates.

### Cell viability assay

**Fig. 1:**
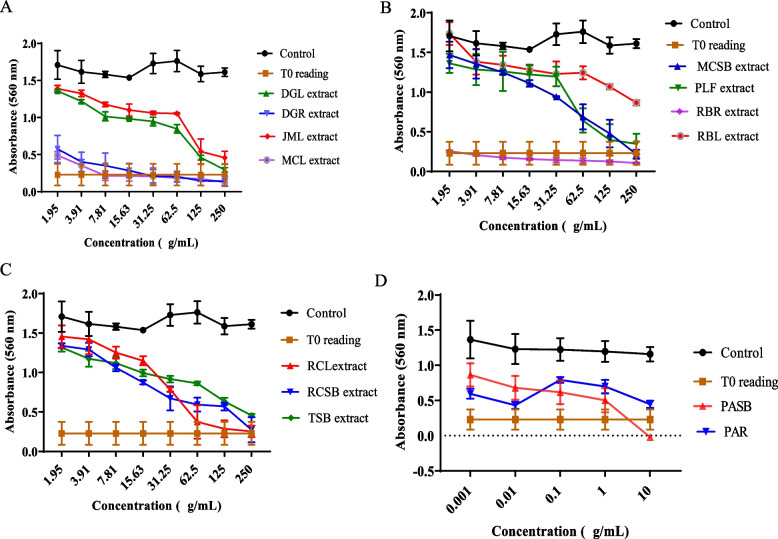
Representative dose-response profiles showing growth inhibitory effect of extracts on MCF-7 cells after 72 hours of treatment, determined by MTT assay (**A**-**D**). Data are expressed as mean ± SD (*n* = 6), *p < 0.05, versus control (a two-way ANOVA followed by Tukey’s post hoc multiple comparison tests)

**Fig. 2:**
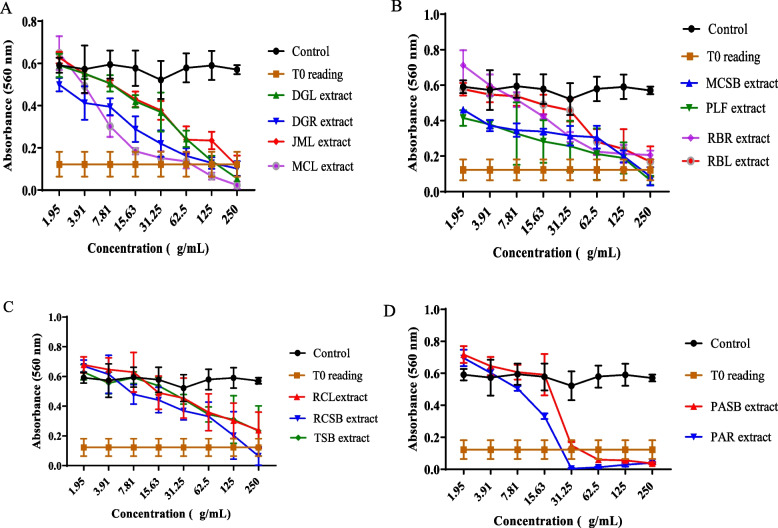
Representative dose-response profiles showing growth inhibitory effect of extracts on HUH-7 cells after 72 hours of treatment, determined by MTT assay (**A**-**D**). Data are expressed as mean ± SD (*n* = 6), *p < 0.05, versus control (a two-way ANOVA followed by Tukey’s post hoc multiple comparison tests)

**Fig. 3:**
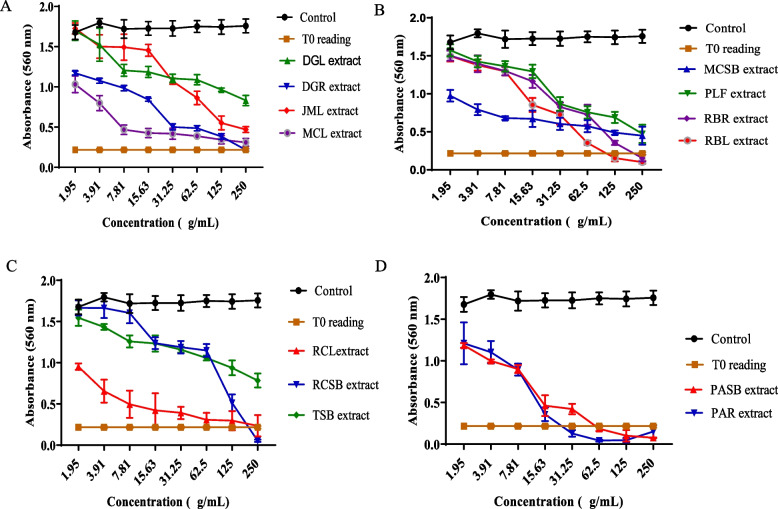
Representative dose-response profiles showing growth inhibitory effect of extracts on HeLa cells after 72 hours of treatment, determined by MTT assay (**A**-**D**). Data are expressed as mean ± SD (*n* = 6), *p < 0.05, versus control (a two-way ANOVA followed by Tukey’s post hoc multiple comparison tests)

The results of the MTT assay show that most of the extracts demonstrated significant growth inhibitory effects on the cell lines used at varying degrees. On the MCF-7 cell line, seven extracts including DGR, MCL, PAR, PASB, RBR, RCL, RCSB exerted antiproliferative activity corresponding to 4.85, 3.42, 0.1, 0.36, 5.88, 30.13 and 20.81 𝜇g/mL, respectively. The extracts of DGR, MCL, PAR, PASB, and PLF produced growth inhibitory effects against the HUH-7 cell line with GI_50_ values of 24.19, 11.54, 16.70, 25.04, and 22.85 𝜇g/mL, respectively. Furthermore, nine extracts including DGR, MCL, MCSB, PAR, PASB, PLF, RBR, RBL, and RCL also demonstrated potent growth inhibitory activity against HeLa cells with GI_50_ values of 10.5, 5.23, 2.87, 7.78, 6.66, 27.82, 13.55, 27.37, and 4.89 𝜇g/mL. These results are summarized in Table 4 and Figs. 1, 2 and 3 (dose-response profiles).

**Table 4:** Growth inhibitory activity of selected extracts and selectivity index

S/no	Extracts	MCF-7	HUH-7	HeLa	MRC-5	MCF-7	HUH-7	HeLa
		Mean GI_50_ values (μg/mL)^1^				Selectivity index (SI)^2^		
1	DGL	70.73 ± 2.54	51.26 ± 12.05	158.16 ± 40.19	25.25 ± 6.27	0.36	0.49	0.16
2	DGR	4.85 ± 1.54	24.19 ± 6.58	10.5 ± 2.07	34.53 ± 1.55	7.12	1.43	3.29
3	JML	99.74 ± 7.18	50.86 ± 3.63	46.99 ± 13.05	134.71 ± 8.86	1.35	2.65	2.87
4	MCL	3.42 ± 1.80	11.54 ± 2.82	5.23 ± 0.37	48.77 ± 6.53	14.26	4.23	9.6
5	MCSB	50.05 ± 3.56	97.14 ± 2.13	2.87 ± 0.90	23.60 ± 6.66	0.47	0.24	8.22
6	PAR	0.06 ± 0.02	16.70 ± 5.04	7.78 ± 0.91	2.89 ± 0.20	48.17	0.17	0.37
7	PASB	0.36 ± 0.13	25.04 ± 2.94	6.66 ± 1.67	2.20 ± 0.13	6.11	0.09	6.11
8	PLF	53.47 ± 0.71	22.85 ± 4.81	27.82 ± 3.25	101.13 ± 30.89	1.89	4.43	3.64
9	RBR	5.88 ± 0.82	43.22 ± 11.16	13.55 ± 1.53	10.51 ± 1.20	1.79	0.24	0.78
10	RBL	> 250	98.26 ± 27.57	27.37 ± 4.75	41.23 ± 14.80	NA	0.42	1.51
11	RCL	30.13 ± 0.15	166.48 ± 9.28	4.89 ± 0.49	91.02 ± 10.44	3.02	0.55	3.02
12	RCSB	20.81 ± 0.57	42.68 ± 10.43	74.46 ± 9.90	56.37 ± 5.28	2.71	1.32	0.76
13	TSB	78.86 ± 2.93	178.70 ± 43.43	140.35 ± 8.52	95.44 ± 20.24	1.21	0.54	0.68
14	Quercetin	2.39 ± 0.74	8.04 ± 0.31	6.09 ± 1.60	6.09 ± 1.60	2.55	0.76	1.00

^1^GI_50_ values are presented as the mean GI_50_ ± SD (μg/mL) of at least three independent experiments. ^2^SI = (GI_50_ of MRC-5)/(GI_50_ of MCF-7, HUH-7 or HeLa).There was a significant difference in growth inhibition in extract-treated cultures compared with DMSO-control treated. *p* < 0.05, versus control (a two-way ANOVA followed by Tukey’s post hoc multiple comparison tests)

### Selectivity index (SI)

All extracts showed greater specificity towards cancer cells at varying degrees. For instance, results showed that MCL, DGR, PASB and RBR extracts exhibited 14-, 7-, 6- and ~ 2-fold selectivity toward MCF-7 cancer cells relative to normal lung fibroblast (MRC-5), respectively. Of note is the extract of MCL, which was 14 times more selective towards MCF-7 cells. These results are also included in Table 4 and supplementary data in Fig. S1.

### Cell count assay

To further assess the effect of selected extracts on cell growth, all cancer cell lines (MCF-7, HeLa, and HUH-7 cells) were subjected to viable cell count using a hemocytometer. Viable cells were counted after each extract treatment at three different concentrations of 0.1, 1, and 10 μg/mL for PASB and PAR (in MCF-7 cell lines only) and 1.95, 15.63, and 125 μg/mL for all other extracts. Results obtained revealed a significant decrease (*p* < 0.0001) in the number of viable cells as concentration increases (Supplementary Fig. S2-S4) compared to control. In the results, HeLa cells treated with control (0.1% DMSO) had 6.9 × 10^5^ cells/ml at the end of the 72 h treatment period whereas PASB extract decreased cell growth to 1.2 × 10^5^, 0.8 × 10^5^, and 0.1 × 10^5^ cells/ml at 1.95, 15.63 and 125 μg/mL, respectively. This same extract decreased the growth of HUH-7 cells to 0.7 × 10^5^, 0.5 × 10^5^, and 0.3 × 10^5^ cells/mL at 1.95, 15.63, and 125 μg/mL, respectively compared to control (1.1 × 10^5^ cells/mL). With MCF-7 cells, PASB extract decreased viable cell number to 0.8 × 10^5^, 0.5 × 10^5^, and less than 0.1 at 0.1, 1, and 10 μg/mL relative to control (1.1 × 10^5^ cells/mL). These results support the MTT assay data as a dose-dependent inhibition of cell growth and proliferation was produced by all extracts tested.

### Colony forming assay

**Fig. 4:**
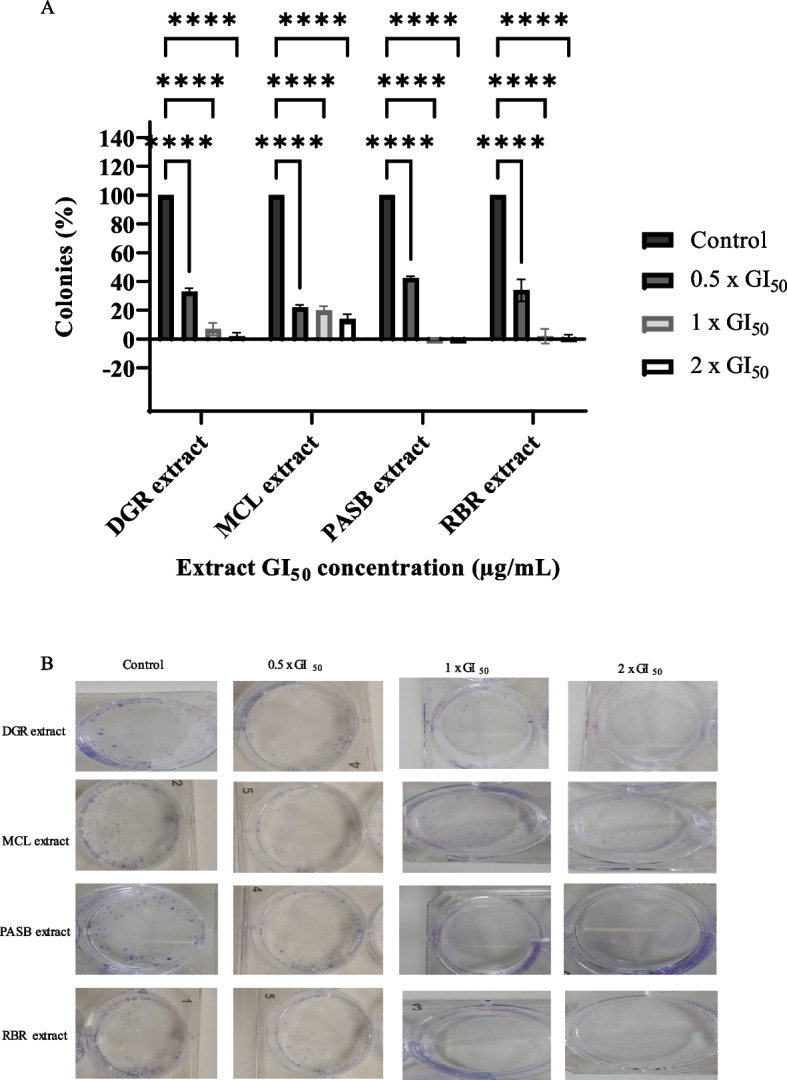
Ethanol extracts of DGR, MCL, PASB, and MCL suppresses colony formation in breast cancer cells at concentrations of 0.5 x GI_50_, 1 x GI_50,_ and 2 x GI_50_. Bars (error bars = SD) denote the mean ± SD of the percentage of colonies formed after exposure to the extracts from three independent determinations (*n* = 3). ^****^p < 0.0001 versus the vehicle-treated control group (**A**). Representative images of plates where ethanol extracts of DGR, MCL, PASB, and MCL suppress colony formation in breast cancer cells at concentrations of 0.5 x GI_50_, 1 x GI_50,_ and 2 x GI_50_ (**B**)

DGR, MCL, PASB, and RBR significantly (*p* < 0.0001) inhibited colony formation in all concentrations tested compared to control (Fig. 4 and Supplementary Fig. S5-S8). There was a decrease in the colony-forming capacity of the extracts with increasing concentration. Remarkable inhibition of colony formation was observed with PASB and RBR extracts at 1 x GI_50_ and 2 x GI_50_ where fewer than 5% of colonies were formed. As for the extracts of DGR and MCL a dose-dependent inhibition of colony formation was also observed at 1 x GI_50_ and 2 x GI_50_ concentrations, respectively (DGR: 7.95 and 2.89%; MCL: 20.4 and 14.26%), relative to control.

### Cell migration assay

**Fig. 5:**
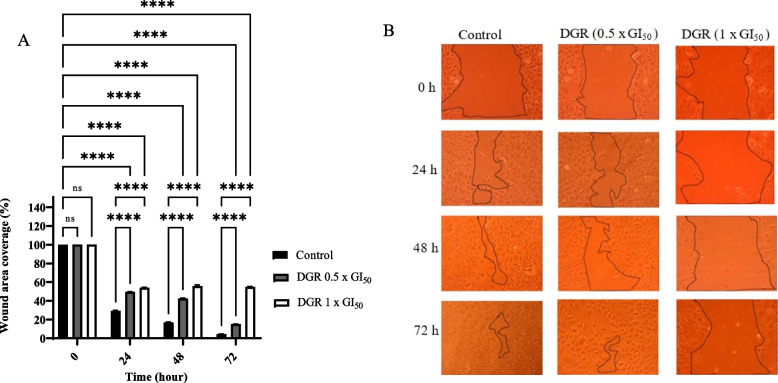
*Dialium guineense* root extract (DGR) significantly inhibited the migration of MCF-7 cells after 24, 48 and 72 h. (**A**) Cell migration was calculated and expressed as the percentage of wound area covered by the cells to the initial cell-free wound area after treatment with either solvent control (0.1% DMSO) or DGR at concentrations of 0.5 x GI_50_ and 1 x GI_50._ The assay was repeated at least three different times. Bar and error bars represent mean ± SD. ****p < 0.0001 vs control. (**B**) The representative images of the migratory cells were taken under an inverted microscope at 10X objective

**Fig. 6:**
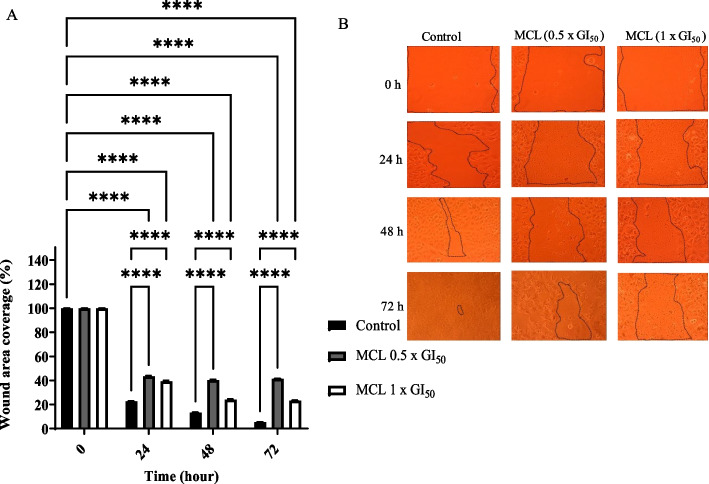
*Musanga cecropioides* leaves extract (MCL) significantly inhibited the migration of MCF-7 cells after 24, 48, and 72 h. (**A**) Cell migration was calculated and expressed as the percentage of the “wound” area covered by the cells to the initial cell-free “wound” area after treatment with either solvent control (0.1% DMSO) or MCL at concentrations of 0.5 x GI_50_ and 1 x GI_50._ The assay was repeated at least three different times. Bar and error bars represent mean ± SD. ****p < 0.0001 vs control. (**B**)The representative images of the migratory cells were taken under an inverted microscope at 10X objective

**Fig. 7:**
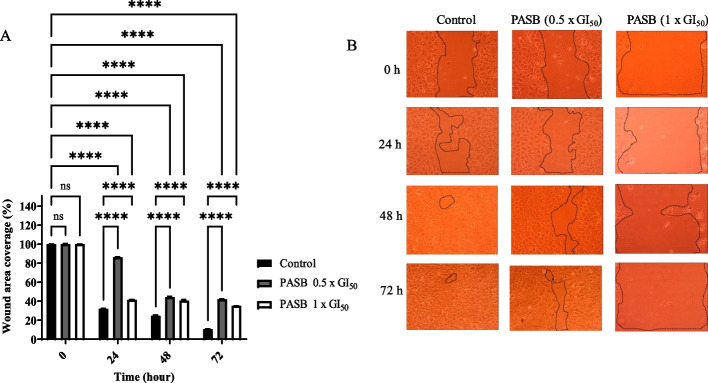
*Piptadeniastrum africanum* stem bark extract (PASB) significantly inhibited the migration of MCF-7 cells after 24, 48 and 72 h. (**A**) Cell migration was calculated and expressed as the percentage of the “wound” area covered by the cells to the initial cell-free “wound” area after treatment with either solvent control (0.1% DMSO) or PASB at concentrations of 0.5 x GI_50_ and 1 x GI_50._ The assay was repeated at least three different times. Bar and error bars represent mean ± SD. ****p < 0.0001 vs control. (**B**)The representative images of the migratory cells were taken under an inverted microscope at 10X objective

**Fig. 8:**
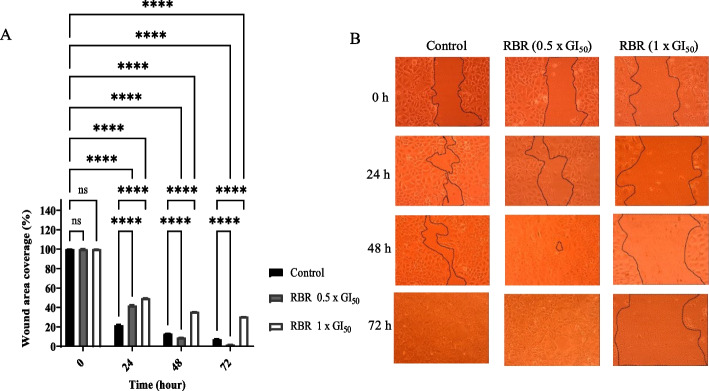
*Raphiostylis beninensis* root extract (RBR) significantly inhibited the migration of MCF-7 cells after 24, 48 and 72 h. (**A**) Cell migration was calculated and expressed as the percentage of the “wound” area covered by the cells to the initial cell-free “wound” area after treatment with either solvent control (0.1% DMSO) or RBR at concentrations of 0.5 x GI_50_ and 1 x GI_50._ The assay was repeated at least three different times. Bar and error bars represent mean ± SD. ****p < 0.0001 vs control. (**B**) The representative images of the migratory cells were taken under an inverted microscope at 10X objective

The cell migration assay is important in evaluating the effect of the selected extracts on the cell matrix as well as the interaction between cells. After scratching and incubating cells for 24, 48, and 72 h, there was still a significant gap in the wound created by extract-treated cells, while the wound coverage area was almost completely closed in the control cells compared to 0 h. For instance, the wound size for DGR extract at 1 x GI_50_ only reduced to 53.89, 55.74 and 54.89%, after 24, 48, and 72 h respectively. When the concentration was lowered (0.5 x GI_50_) wound size reduced to 49.51, 42.52 and 15.01% after 24, 48, and 72 h, respectively which was still significantly higher (*p* < 0.0001) compared to the control where the wound sizes were 29.29, 16.95 and 4.39% at 24, 48 and 72 h, respectively. Figures 5, 6, 7 and 8 demonstrate the anti-migratory ability of the extracts of DGR, MCL, PASB, and RBR in MCF-7 cells compared to control in a dose and time-dependent manner.

### Polyphenolic content determination

Table 5 presents the total phenolics, flavonoids, and proanthocyanidins contents estimated in MCL, DGR, PASB, and RBR extracts. These extracts exhibited total phenolic content of 37.79, 5.77, 46.34, 19.18 of gallic acid equivalent per one gram of dried extracts respectively; total flavonoid contents were 12.84, 1.90, 2.73, 2.67 of quercetin equivalent per one gram of dried extracts respectively while the total condensed tannins were estimated to be 183.61, 11.87, 54.30, 96.19 of catechin equivalent per one gram of dried extracts respectively.

**Table 5:** Polyphenolic content of *M. cecropioides, D. guineense, P. africanum* and *R. beninensis*

Extract	Total phenolics^a^	Total flavonoids^b^	Total proanthocyanidins^c^
MCL	37.79 ± 10.54	12.84 ± 0.31	183.61 ± 7.10
DGR	5.77 ± 0.61	1.90 ± 0.14	11.87 ± 1.85
PASB	46.34 ± 0.97	2.73 ± 1.50	54.30 ± 0.13
RBR	19.18 ± 5.03	2.67 ± 0.22	96.19 ± 1.07

Data are expressed as mean ± SEM (*n* = 3) of three independent determinations. ^a^, expressed as mg gallic acid/g of dried extract; ^b^, expressed as mg quercetin/g of dried extract; ^c^, expressed as mg catechin/g of dried extract.

## Discussion

Over three thousand medicinal plant species have been reported to exert significant cytotoxic effects against malignancies globally [32]. To this end, phytocompounds derived from them are becoming more widely acknowledged as effective cancer treatments [33]. Furthermore, the emergence of resistance to currently available chemotherapy has heightened the interest of researchers to search for target therapy from natural sources including medicinal plants which contain useful metabolites acting singly or synergistically to improve health while also serving as scaffolds for the development of safer therapeutic agents [34].

The cytotoxic activity of some selected medicinal plants used in Nigerian folk medicine for tumour-related problems was investigated using brine shrimp lethality, MTT, clonogenic, and cell migration assays. Total polyphenolic contents in the active extracts were also estimated.

The brine shrimp lethality assay (BSLA) is a straightforward and inexpensive bioassay employed for the preliminary screening of cytotoxic phytochemicals found in plant extracts [35]. As shown in Table 3, all the extracts exhibited significant toxicity against brine shrimp larvae. The LC_50_ of the extracts PASB, PAR, RBR, and PLF were < 100 μg/mL revealing potent toxicity against brine shrimp larvae while six other extracts including JML, TSB, DGR, MCL, RBL, and RCL produced moderate toxicity (LC_50_: 100–500 μg/mL) against brine shrimp larvae. This indicates the possibility of the presence of potent antitumor compounds in the extracts. The extracts of RCSB and two other extracts including MCSB and DGL produced weak (LC_50_: 500–1000 μg/mL) and no toxicity (LC_50_ > 1000 μg/mL), respectively against brine shrimp larvae. Plants that have proven to be toxic to brine shrimps are promising candidates for anti-cancer research since at higher doses, bioactive compounds are highly toxic, therefore a basic zoological entity can be used to provide quick information in in-vivo lethality screening [36].

The MTT assay is a colorimetric technique that evaluates cell viability based on its sensitivity and reliability. It relies on the ability of mitochondrial dehydrogenase enzyme in viable cells to convert the yellow water-soluble substrate 3-(4,5-dimethylthiazol-2yl)-2,5-diphenyl tetrazolium bromide (MTT) into a dark purple formazan product that is water-insoluble, and the amount of formazan produced is related to the number of cells present [37].

The American Cancer Institute recommends a minimum growth inhibitory concentration (GI_50_) of ≤30 μg/mL after 72 hours of exposure to any cancer cell line for an extract to be considered a potential cytotoxic candidate. Similarly, a crude plant extract with a IC_50_ of ≤20 μg/ mL, is regarded as highly cytotoxic [38, 39]. From the results, the extracts of DGR, MCL, PASB, RBR, RCL, RCSB exhibited potent cytotoxic effects against the breast cancer cell line in a dose-dependent manner thereby meeting the criteria with GI_50_ values ≤30 μg/mL. The extract of PAR was also active against MCF-7 cells, the effect was however not dose dependent. The extracts of DGR, MCL, PAR, PASB, and PLF also produced significant cytotoxic activity against the HUH-7 cancer cell line. Regarding HeLa cells, more extracts including DGR, MCL, MCSB, PAR, PASB, PLF, RBR, RBL, and RCSB also demonstrated significant growth inhibitory activity. Similar to the effect produced against MCF-7 cells, the activity of PAR against HeLa cells was also non-dose dependent. The non-dose dependency effect exhibited by PAR extract could be explained using Clarke’s receptor occupancy theory which quantifies the relationship between drug concentration and response as being linear [40]. The behaviour of this extract showed that the sensitivity of the extract caused inhibition in cell viability to occur at physiologically relevant concentrations (low concentrations) and increasing concentrations resulted in no further inhibition in cell viability. This effect is consistent with previous studies where similar effects have been reported [41, 42].

Interestingly, the results obtained from the MTT assay corroborates that of the brine shrimp lethality as most of the extracts that showed cytotoxic activity against brine shrimps were also active against the different cancer cell lines studied.

The cell counts results obtained following 72 h extract exposure to cells corroborated the growth inhibitory effect detected by the MTT assay earlier conducted as a significant decrease (*p* < 0.0001) in the number of viable cells was observed as the extract concentration increased. This implies that inhibition of cell growth and proliferation occurred in a dose-dependent manner in the majority of the selected extracts which is indicative of the cytostatic or cytotoxic activity of the extracts.

Selectivity of extracts towards cancer cells while exhibiting minimal toxicity towards normal cells is crucial since the goal of cancer chemotherapy is to specifically target cancer cells. This lack of molecular target therapy is associated with several chemotherapeutic agents [43]. Hence, the specificity of plant extracts toward cancer cells was evaluated using normal human lung fibroblast (MRC-5) cells. According to selectivity index classification, an SI index less than 1 is classified as non-selective, between 1 and 10 is weakly selective and an SI value above 10 is regarded safe (non-toxic) [2]. Based on overall results, MCL extract was more active against MCF-7 than HeLa cells and HUH-7 cells. It exhibited superior cytotoxic activity compared to quercetin (positive control) and demonstrated 14-fold cancer selectivity towards MCF-7 cells. Other extracts including DGR, PASB, PAR, RCL, JML, TSB, and MCSB also showed significant selectivity against all cancer cell lines at varying degrees (Table 4).

The clonogenic or colony-forming assay is considered a gold standard that evaluates the ability of single cancer cells to survive and grow into colonies (more than 50 cells) after a brief exposure to test agents [44]. To investigate the cytotoxic effect of MCL, DGR, PASB, and RBR on colony formation, a clonogenic assay was carried out using MCF-7 cells after 24 h of treatment. After 10 days of incubation, results showed that all extracts significantly inhibited colony formation in a dose-dependent manner compared to control. This is suggestive of the ability of the extracts to cause the cells to lose their proliferative potential and prevent tumour recolonization.

Cell migration is an important process in which cells must be able to shift and reach their proper position in any environment to carry out their functions. This process is crucial in many biological processes including tumour invasion and metastasis [45]. The effect of MCL, DGR, PASB, and RBR extracts on the migration of MCF-7 cells was investigated. Results revealed that migration of MCF-7 cells was reduced by ~ 2-fold at 1 x GI_50_ and 0.5 x GI_50_ by DGR extracts relative to control after 24, 48 and 72 h. The other three extracts acted in a similar manner indicating the significant inhibitory effect on migration. These results and those of the clonogenic assay signal the potential of the investigated extracts to decrease and inhibit proliferation, invasion, and metastasis in cancer cases.

Nowadays, the demand for plants rich in polyphenolic compounds is on the rise owing to their health benefits and based on their ability to reduce lipid peroxidation in oxidative stress-related diseases including cancer [46]. Some authors have reported a correlation of considerable levels of polyphenolic content in medicinal plants with anticancer activities [47]. They can inhibit or alter the regulation of proteins and other agents that may be contributing to the survival of cancer cells. Proteins such as Signal Transducers and Activator of Transcription (STAT) are anti-apoptotic and contribute to cancer cell growth. Polyphenolics, especially flavonoids inhibit the expression of NF-κB necessary for cancer cell survival, angiogenesis, and proliferation [48]. The crude extracts of MCL exhibited the highest proanthocyanidins and flavonoid contents while PASB extract demonstrated the highest phenolic content. In addition to the bioactive secondary metabolites, the ability of these extracts to inhibit cell proliferation, colony formation, and cell migration indicates that they may be useful therapeutic agents for tumour-related diseases.

## Conclusion

The findings of this study support the view that medicinal plants are promising sources of potential cytotoxic agents that may be effective for cancer therapy. In this study, the extracts of MCL, DGR, PASB, and RBR exhibited remarkable antiproliferative potential while also showing profound selectivity to breast cancer cell line. These extracts also demonstrated significant inhibitory colony forming and cell migration ability. However, further detailed mechanistic studies coupled with bio-assay guided purification in a bid to isolate, identify, and characterize the active cytotoxic compounds from these plants are ongoing and will be reported in due course.

### Supplementary Information

**Additional file 1: Figure S1** Graph showing the selectivity indices of six active extracts against MCF-7 and HeLa cells. **Figure S2** Cell count assay of MCF-7 cells after 72 h extracts exposure. Data are expressed as mean±SD (n=3), ****p<0.0001, versus control (a two-way ANOVA followed by Tukey’s *post hoc* multiple comparison tests). **Figure S3** Cell count assay of HUH-7 cells after 72 h extracts exposure. Data are expressed as mean±SD (n=3), ****p<0.0001, versus control (a two-way ANOVA followed by Tukey’s *post hoc* multiple comparison tests). **Figure S4** Cell count assay of HeLa cells after 72 h exposure. Data are expressed as mean±SD (n=3), ****p<0.0001, versus control (a two-way ANOVA followed by Tukey’s *post hoc* multiple comparison tests). **Figure S5** Representative images of plates where ethanol extracts of DGR suppresses colony formation in breast cancer cells at concentrations of 0.5 x GI_50_, 1 x GI_50_ and 2 x GI_50_. **Figure S6** Representative images of plates where ethanol extracts of MCL suppresses colony formation in breast cancer cells at concentrations of 0.5 x GI_50_, 1 x GI_50_ and 2 x GI_50_. **Figure S7** Representative images of plates where ethanol extracts of PASB suppresses colony formation in breast cancer cells at concentrations of 0.5 x GI_50_, 1 x GI_50_ and 2 x GI_50_. **Figure S8** Representative images of plates where ethanol extracts of RBR suppresses colony formation in breast cancer cells at concentrations of 0.5 x GI_50_, 1 x GI_50_ and 2 x GI_50_.

## Data Availability

All datasets generated during this study are available upon reasonable request from the corresponding author.

## Abbreviations

MTT: 3-(4,5-dimethylthiazol-2-yl)-2,5-diphenyltetrazolium bromide (MTT)
MCF-7: Breast cancer
HeLa: Cervical cancer
HUH-7: Liver cancer
MRC-5: Normal lung fibroblast
LC_50_: 50% lethal concentration
IC_50_: 50% inhibitory concentration
GI_50_: 50% growth inhibitory concentration
DGR: *Dialium guineense* root
DGL: *Dialium guineense* leaves
JML: *Jateorhiza macrantha* leaves
MCL: *Musanga cecropioides* leaves
MCSB: *Musanga cecropioides* stembark
PASB: *Piptadeniastrum africanum* stembark
PAR: *Piptadeniastrum africanum* root
PLF: *Pupalia lappacea* flower tops
RBR: *Raphiostylis beninensis* root
RBL: *Raphiostylis beninensis* leaves
RCL: *Ritchiea capparoides* leaves
RCSB: *Ritchiea capparoides* stembark
TSB: *Triplochiton scleroxylon* stembark
AlCl_3_: Aluminium chloride
DMSO: Dimethyl sulfoxide
DMEM: Dulbecco’s Modified Eagle Medium
NF-κB: nuclear factor kappa B cells
GAE: Gallic acid equivalent.
