# Supplementary material for: Antiproliferative activities of some selected Nigerian medicinal plants against breast, liver, and cervical cancer cells
Source: BMC Complement Med Ther. 2024 Mar 6;24:110. doi: 10.1186/s12906-024-04365-w (PMC10916328; doi:10.1186/s12906-024-04365-w)
Supplement: Supplementary file 1 — Additional file 1: Figure S1 Graph showing the selectivity indices of six active extracts against MCF-7 and HeLa cells. Figure S2 Cell count assay of MCF-7 cells after 72 h extracts exposure. Data are expressed as mean±SD (n=3), ****p<0.0001, versus control (a two-way ANOVA followed by Tukey’s post hoc multiple comparison tests). Figure S3 Cell count assay of HUH-7 cells after 72 h extracts exposure. Data are expressed as mean±SD (n=3), ****p<0.0001, versus control (a two-way ANOVA followed by Tukey’s post hoc multiple comparison tests). Figure S4 Cell count assay of HeLa cells after 72 h exposure. Data are expressed as mean±SD (n=3), ****p<0.0001, versus control (a two-way ANOVA followed by Tukey’s post hoc multiple comparison tests). Figure S5 Representative images of plates where ethanol extracts of DGR suppresses colony formation in breast cancer cells at concentrations of 0.5 x GI50, 1 x GI50 and 2 x GI50. Figure S6 Representative images of plates where ethanol extracts of MCL suppresses colony formation in breast cancer cells at concentrations of 0.5 x GI50, 1 x GI50 and 2 x GI50. Figure S7 Representative images of plates where ethanol extracts of PASB suppresses colony formation in breast cancer cells at concentrations of 0.5 x GI50, 1 x GI50 and 2 x GI50. Figure S8 Representative images of plates where ethanol extracts of RBR suppresses colony formation in breast cancer cells at concentrations of 0.5 x GI50, 1 x GI50 and 2 x GI50. [file 12906_2024_4365_MOESM1_ESM.docx]

**Supplementary data for antiproliferative potential of some selected Nigerian Medicinal plant by Olaleye et al 2023**

Figure S1: Graph showing the selectivity indices of six active extracts against MCF-7 and HeLa cells

A

B

C

D

Figure.S2: Cell count assay of MCF-7 cells after 72 h extracts exposure. Data are expressed as mean±SD (n=3), ****p<0.0001, versus control (a two-way ANOVA followed by Tukey’s *post hoc* multiple comparison tests).

A

B

C

D

Figure S3: Cell count assay of HUH-7 cells after 72 h extracts exposure. Data are expressed as mean±SD (n=3), ****p<0.0001, versus control (a two-way ANOVA followed by Tukey’s *post hoc* multiple comparison tests).

B

A

D

C

Figure S4: Cell count assay of HeLa cells after 72 h exposure. Data are expressed as mean±SD (n=3), ****p<0.0001, versus control (a two-way ANOVA followed by Tukey’s *post hoc* multiple comparison tests).


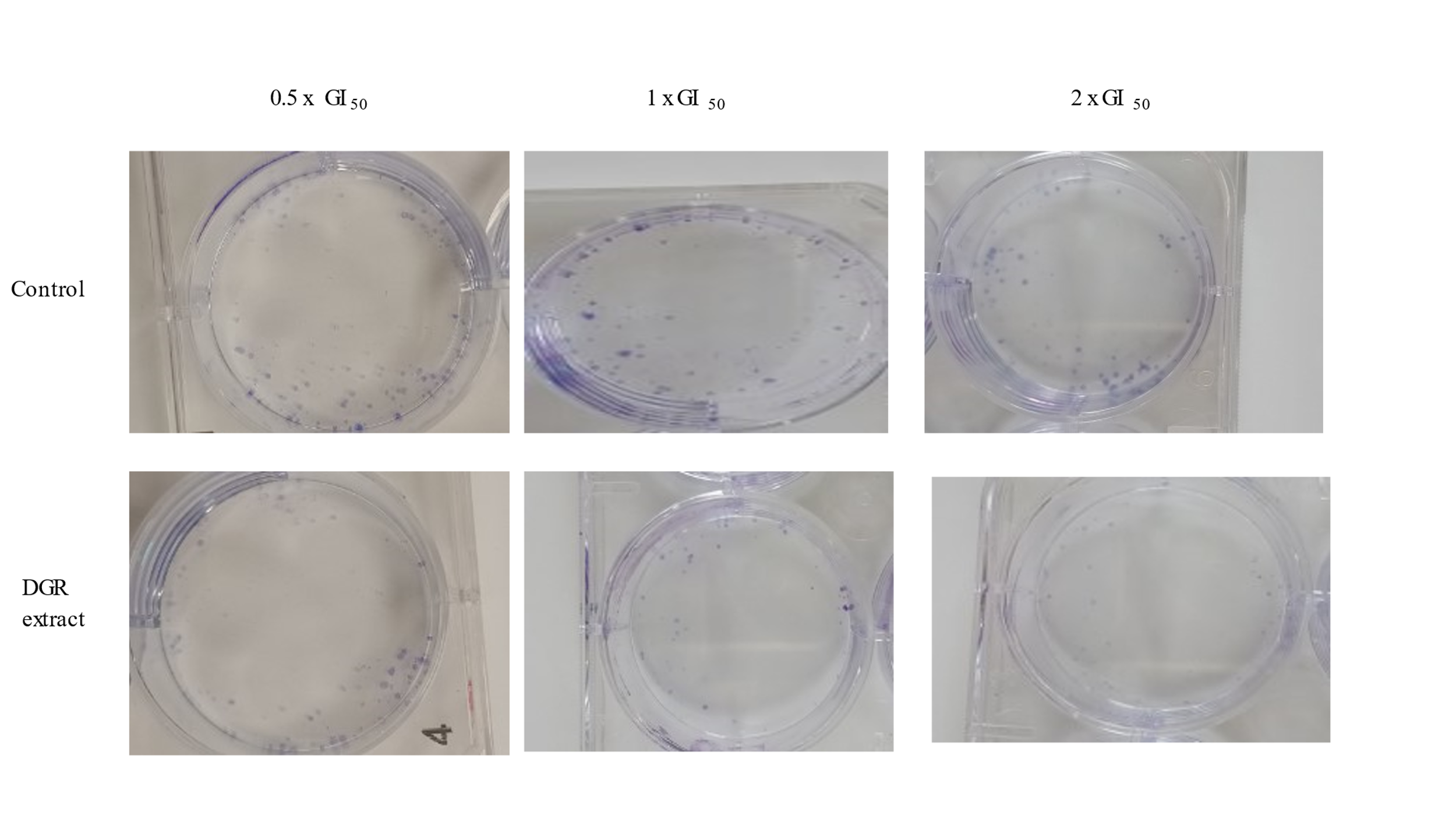


Figure S5: Representative images of plates where ethanol extracts of DGR suppresses colony formation in breast cancer cells at concentrations of 0.5 x GI_50_, 1 x GI_50_ and 2 x GI_50_.


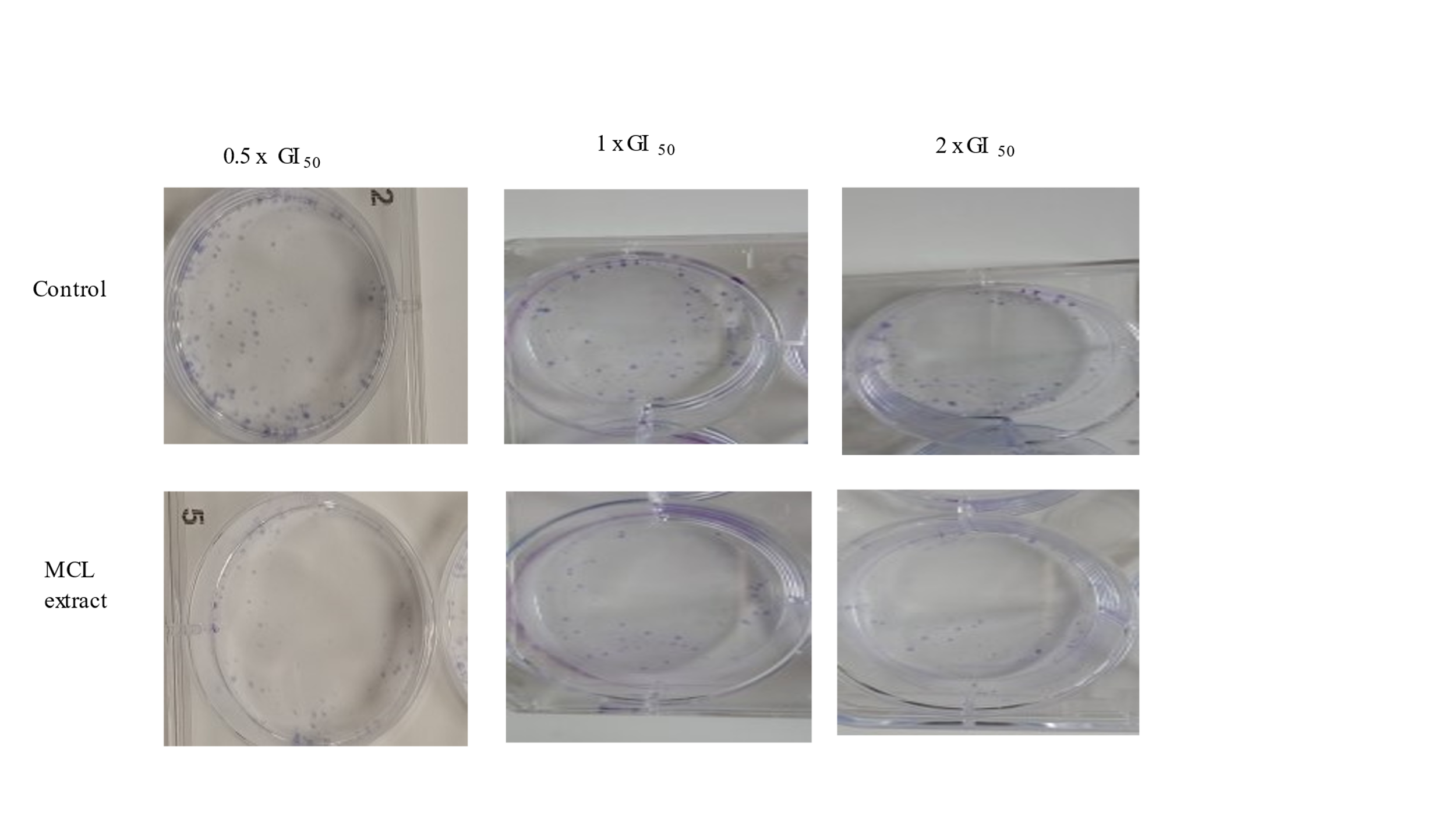


Figure S6: Representative images of plates where ethanol extracts of MCL suppresses colony formation in breast cancer cells at concentrations of 0.5 x GI_50_, 1 x GI_50_ and 2 x GI_50_.


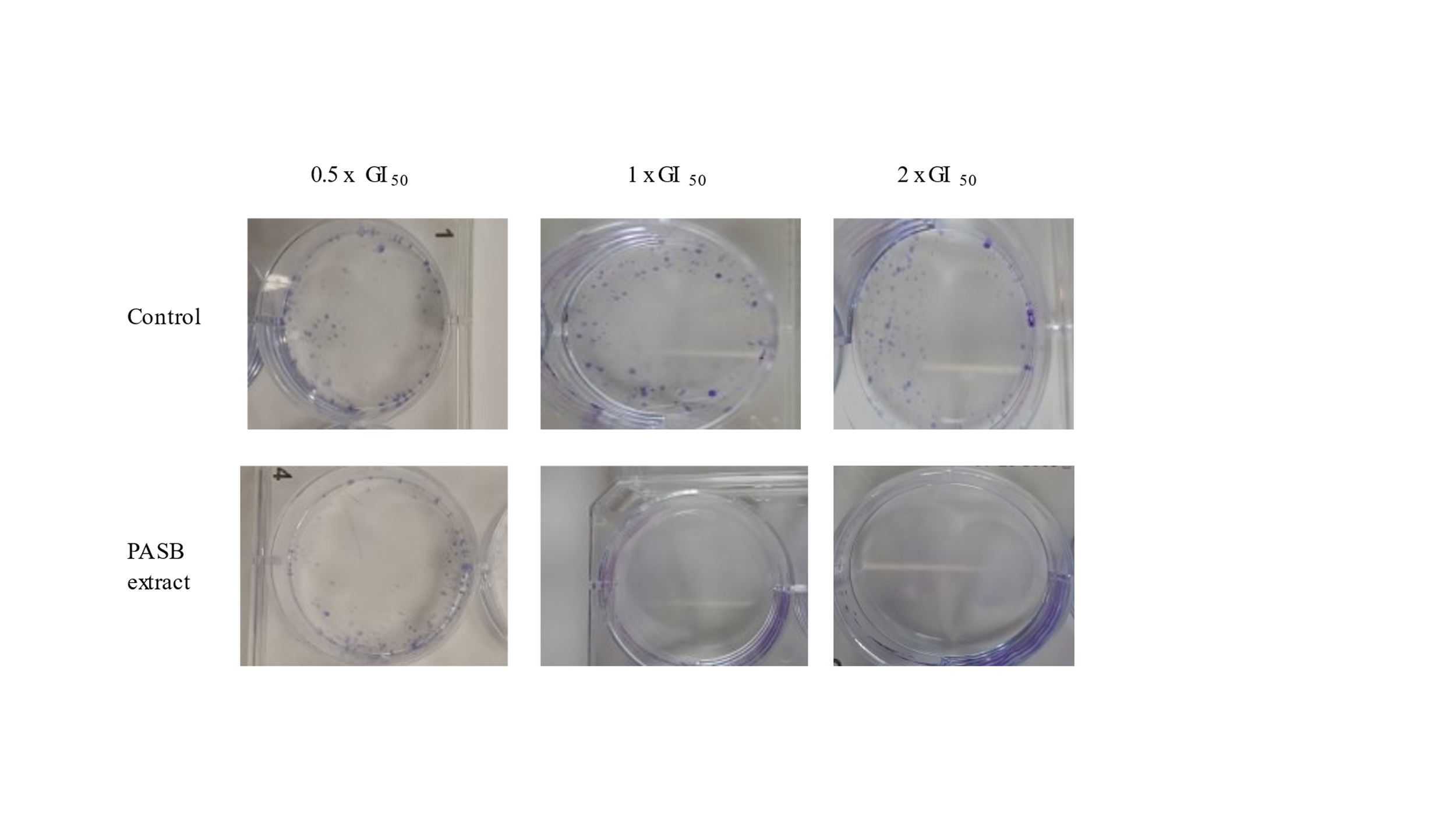


Figure S7: Representative images of plates where ethanol extracts of PASB suppresses colony formation in breast cancer cells at concentrations of 0.5 x GI_50_, 1 x GI_50_ and 2 x GI_50_.


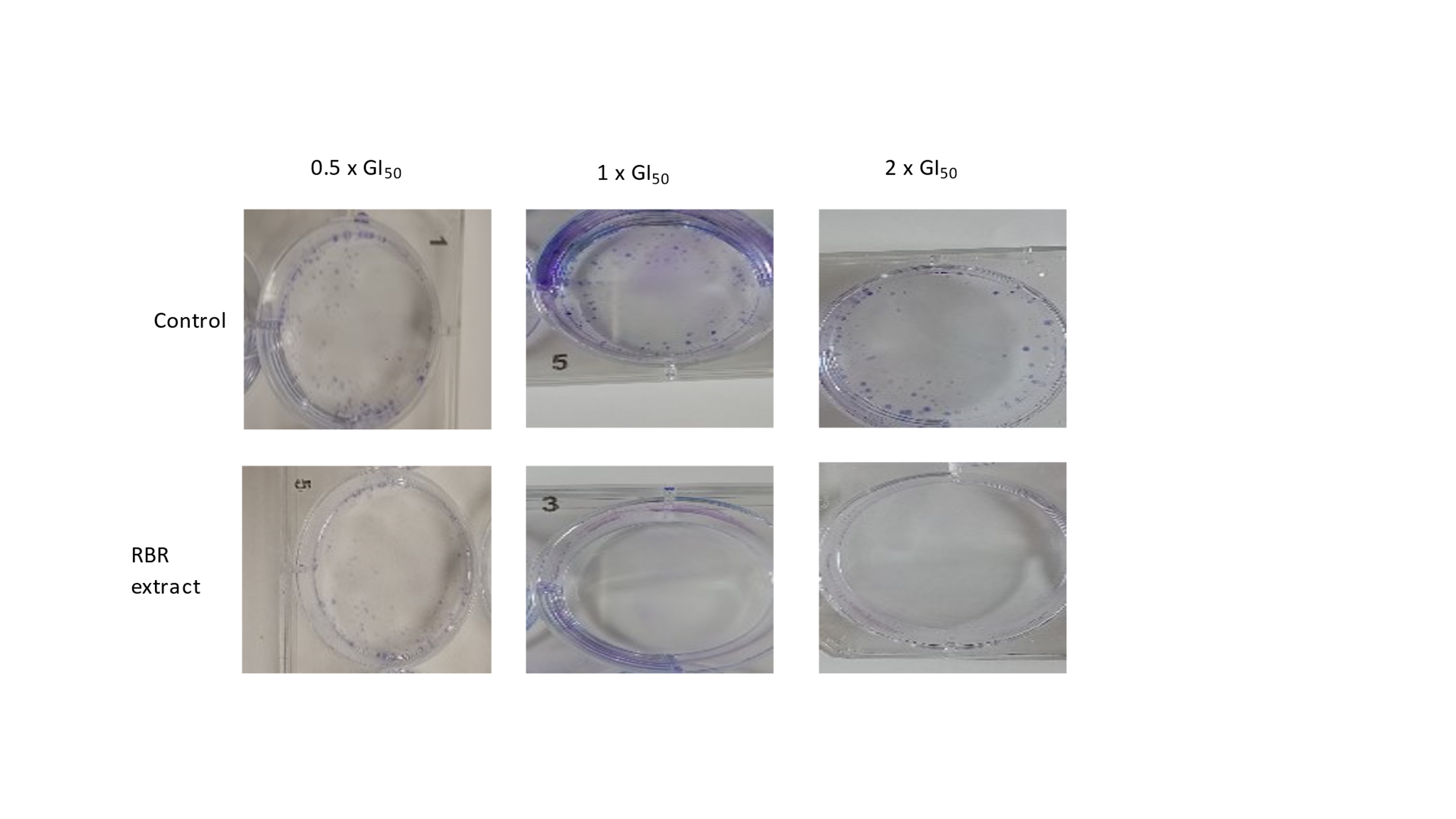


Figure S8: Representative images of plates where ethanol extracts of RBR suppresses colony formation in breast cancer cells at concentrations of 0.5 x GI_50_, 1 x GI_50_ and 2 x GI_50_.
